# Repeatability of Metabolic Imaging With Hyperpolarized Pyruvate: Back‐to‐Back Neuroimaging and Blood Analysis

**DOI:** 10.1002/mrm.70402

**Published:** 2026-04-23

**Authors:** Jun Chen, Sung‐Han Lin, Jessica Sudderth, Kelley A. Derner, Crystal E. Harrison, Maheen Zaidi, Jeannie D. Baxter, Jeff Liticker, Zohreh Erfani, Craig R. Malloy, Marco C. Pinho, Ralph J. DeBerardinis, Jae Mo Park

**Affiliations:** ^1^ Advanced Imaging Research Center, the University of Texas Southwestern Medical Center Dallas Texas USA; ^2^ Children's Research Institute, the University of Texas Southwestern Medical Center Dallas Texas USA; ^3^ Department of Radiology The University of Texas Southwestern Medical Center Dallas Texas USA; ^4^ Howard Hughes Medical Institute Dallas Texas USA; ^5^ McDermott Center for Human Growth and Development, the University of Texas Southwestern Medical Center Dallas Texas USA; ^6^ Department of Biomedical Engineering The University of Texas Southwestern Medical Center Dallas Texas USA; ^7^ Charles and Jane Pak Center for Mineral Metabolism and Clinical Research, the University of Texas Southwestern Medical Center Dallas Texas USA

**Keywords:** hyperpolarized pyruvate, lactate clearance, neuroimaging, repeatability

## Abstract

**Purpose:**

^13^C MRI with hyperpolarized [1‐^13^C]pyruvate enables noninvasive imaging of metabolic pathways. Considering the high dose of pyruvate used in hyperpolarized ^13^C‐pyruvate studies, circulating ^13^C‐pyruvate and the resulting ^13^C‐lactate in plasma may influence subsequent pyruvate metabolism, yet the guidance on consecutive injections does not exist. This study is to characterize blood pyruvate and lactate dynamics following [1‐^13^C]pyruvate injection and to evaluate the repeatability of ^13^C neuroimaging with consecutive pyruvate injections.

**Methods:**

Sixteen healthy adults (mean age, 39.0 ± 15.9 years; 8 men) underwent blood sampling or MRI. Eight participants received bolus injections of non‐hyperpolarized [1‐^13^C]pyruvate following both overnight fasting and postprandial states. Blood samples were collected at baseline and up to 60 min post‐injection to quantify pyruvate and lactate concentrations and ^13^C fractional enrichments using mass spectrometry. In parallel, eight participants underwent ^13^C/^1^H MRI that included two injections of hyperpolarized [1‐^13^C]pyruvate with intervals of 6–55 min for time‐resolved measurement of ^13^C‐pyruvate and ^13^C‐lactate in the brain.

**Results:**

Plasma ^13^C‐pyruvate peaked within 30 s and returned to baseline within 5–10 min, while ^13^C‐lactate peaked at 2–3 min. Lactate ^13^C enrichment was higher in fasting versus fed states, and total lactate concentration in blood was unchanged by pyruvate injection. Brain ^13^C‐lactate/pyruvate showed excellent repeatability (ICC = 0.9842), with all points within the 95% Bland–Altman limits. Voxel‐wise Pearson's correlation between injections was 0.72 ± 0.22 and within‐subject coefficient of variation was 17.6% ± 4.6%.

**Conclusion:**

Consecutive injections of hyperpolarized [1‐^13^C]pyruvate yield repeatable blood and brain metabolic measurements in humans, supporting multi‐injection protocols in ^13^C MRI studies.

## 1 Introduction

^13^C MRI with hyperpolarized (HP) [1‐^13^C]pyruvate visualizes metabolic fate of pyruvate toward anaerobic and oxidative pathways in vivo, offering a noninvasive window into metabolic dysfunction. Because ^13^C is a stable isotope, repeated administrations of ^13^C‐pyruvate are possible without radiation exposure unlike positron emission tomography (PET) tracers. The typical human dose of HP pyruvate is approximately 0.1 mmol/kg body weight, which is orders of magnitude larger in mass than PET tracers, where injected masses are in the microgram to sub‐microgram range [1]. Adult PET scans using [^18^F]fluorodeoxyglucose (FDG) commonly involve activities of ∼200–400 MBq (5–10 mCi), corresponding to tracer masses well below a milligram (i.e., < 0.01–0.1 mg) [2].

The SPINlab, currently the only clinically available polarizer, has been used for all human studies. As the clinical polarizer supports four independent channels, consecutive injections of HP pyruvate are feasible within a single MR session. This capability has been applied in neuro‐ and cardiac‐imaging studies to confirm findings [3, 4, 5, 6, 7], mitigate potential dissolution failures [8, 9, 10], test metabolic interventions [11, 12], or enhance sensitivity by averaging signals [13]. Injection intervals have typically ranged from 20 to 60 min. However, the repeatability of HP pyruvate in humans has not been systematically characterized, and no guidelines exist for multiple injections.

A major concern is the elevation of circulating lactate, which can influence HP [1‐^13^C]pyruvate metabolism through rapid bidirectional isotope exchange between pyruvate and lactate. Hurd et al. demonstrated in rodents that co‐injection of unlabeled lactate with HP [1‐^13^C]pyruvate increased ^13^C exchange between pyruvate and lactate when endogenous lactate pools were elevated [14]. Moreover, elevated lactate levels can downregulate cerebral glycolysis by direct inhibition of phosphofructokinase and subsequent inhibition of hexokinase [15, 16]. Systemic lactate levels are typically higher in the postprandial state due to increased insulin‐driven glycolysis in peripheral tissues, whereas fasting favors reduced glycolytic flux and greater reliance on fatty acid oxidation, resulting in lower baseline lactate [17]. While most HP brain studies have not reported the nutritional status of the study participants, practical clinical scheduling for procedures such as tumor resection [18] and FDG‐PET scans [19] typically requires at least 4–6 h of fasting, as was done in HP glioblastoma patient studies [9]. Thus, characterizing how circulating lactate responds to a pyruvate bolus and how quickly it returns to baseline under both fasted and fed conditions is critical for assessing the repeatability of HP pyruvate exams.

In this study, we characterize the dynamics of blood pyruvate and lactate following [1‐^13^C]pyruvate injection using mass spectrometry from healthy participants and assess the repeatability of HP pyruvate in neuroimaging by performing back‐to‐back injections with intervals ranging from 6 to 55 min.

## 2 Methods

### 2.1 Study Participants

This prospective study was approved by the local Institutional Review Board (STU‐2018‐0227, STU‐2018‐0013, STU‐072017‐009) and conducted between August 2018 and August 2025. Sixteen healthy volunteers were enrolled, with eight subjects participating in blood analyses and eight in imaging studies. Recruitment was performed by word‐of‐mouth. Eight healthy volunteers (age 34.1 ± 15.0 years, BMI 27.4 ± 6.2 kg/m^2^, five women) were enrolled for blood analysis (Table 1). Eight healthy subjects (age = 43.9 ± 16.1 years, BMI = 26.8 ± 2.7 kg/m^2^, five men) were enrolled in the imaging study (Table 2). Three of the participants (#14–16) have been previously reported [3]. This prior article described a methodological alternative to imaging approaches whereas in this manuscript we report on the repeatability of HP pyruvate. Written informed consent was obtained from all participants after the study procedures had been fully explained. The study was HIPAA compliant and conducted under an Investigational New Drug approval by the US Food and Drug Administration (IND 133229).

**TABLE 1:** Demographics of participants for blood study.

ID	Sex	Race	Age (years)	Height (cm)	Weight (kg)	BMI (kg/m^2^)	Blood analysis	Nutritional condition
1	Male	Asian	24	165	75.3	27.6	X	Fast/fed
2	Female	White	24	163	87.2	33.0	X	Fast/fed
3	Male	White	23	180	79.5	24.4	X	Fast/fed
4	Female	White	30	160	53.6	20.9	X	Fast/fed
5	Male	White	33	188	83.2	23.6	X	Fast/fed
6	Female	White	64	163	85.0	32.2	X	Fast/fed
7	Female	White	50	163	98.4	27.3	X	Fed
8	Female	White	25	170	58.9	20.3	X	Fast/fed

**TABLE 2:** Demographics of participants for HP imaging study.

ID	Sex	Race	Age (years)	Height (cm)	Weight (kg)	BMI (kg/m^2^)	HP imaging	Interval between injections (min)
9	Male	White	39	183	86.4	25.8	MISI	6
10	Male	White	33	163	68.2	25.7	MISI	33
11	Male	Black	25	178	111.7	33.2	MISI	35
12	Male	White	63	173	74.84	25.0	spCSI	48
13	Male	Asian	54	170	73.48	25.4	spCSI	46
14	Female	White	48	152	61.24	26.5	MRS	46
15	Female	White	24	152	58.9	25.4	MRS	55
16	Female	White	65	165	75.7	27.8	MRS	44

### 2.2 Blood Collection and Processing

Venous blood was collected in two consecutive sessions separated by 90 min. Except for one participant (#7), all other subjects arrived after an overnight fast. A sterile solution of [1‐^13^C]pyruvate, neutralized to physiological pH with NaOH, was prepared and administered intravenously as a bolus (0.4 mL/kg, injection rate 5 mL/s) using a power injector. Separate intravenous lines were placed for pyruvate injection and blood collection. Baseline samples were obtained prior to injection, followed by serial 1‐mL blood draws at 30, 60, 90, 120 s, 3, 5, 10, 15, 30, 45, and 60 min post‐injection. Between sessions, subjects were provided with a standardized light snack (plain bagel with peanut butter and fruit juice) consumed within 15 min. To minimize post‐collection metabolic changes, blood samples were injected into 20 mL methanol, vortexed, and spun down (13 000 rpm, 4°C, 10 min). A 2 mL of the supernatant was sent for mass spectrometry analysis as previously described [20].

### 2.3 MRI Protocol

All examinations were performed on a wide‐bore 3T MR scanner (Discovery 750w, GE Healthcare; 70‐cm bore; 25 mT/m maximum gradient amplitude, 120 mT/m/ms maximum slew rate). A dual frequency ^13^C/^1^H head coil (Clinical MR Solutions, Brookfield, WI, USA) was used for RF excitation and data acquisition [3].

All study participants ingested 75 g of oral glucose gel approximately 1 h prior to the first HP pyruvate injection to standardize metabolic conditions and promote a consistent post‐prandial state. Each subject underwent an integrated ^13^C/^1^H protocol with two injections of HP [1‐^13^C]pyruvate. After anatomical localization and B_0_ shimming, two identical ^13^C scans were performed without repositioning the subject. Three acquisition schemes were exploited for ^13^C neuroimaging. For participants #12 and #13, dynamic spiral chemical shift imaging (spCSI) was applied to a single brain slice with variable flip angles up to 30° per timepoint and a 5‐s temporal resolution. For participants #9–11, metabolite‐interleaved spiral imaging (MISI) was used to acquire three axial slices every 5.3 s, utilizing asymmetric flip angles (90° for bicarbonate, 90° for lactate, 10° for pyruvate) and a custom‐designed spectral‐spatial RF pulse [4]. For participants #14–16, dynamic MRS with small tip angle was used (temporal resolution = 3 s, #slice = 4, slice thickness = 1.5 cm). Detailed MR protocol and schematics are available in Supporting Information: Methods and Figure S1. Data acquisition began 25 s after the start of injection when MISI was used whereas spCSI and MRS started immediately with the injection. The second ^13^C scan, with an additional injection of HP [1‐^13^C]pyruvate, was performed 6–55 min after the first injection.

### 2.4 Dynamic Nuclear Polarization and Dissolution

A clinical SPINlab polarizer (GE Healthcare) was used for dynamic nuclear polarization. Two pyruvate samples were polarized simultaneously for each subject. Procedures for sample preparation and polarization are described in Supporting Information. The HP pyruvate solution was mixed with 36.5 mL of room temperature TRIS/NaOH media (333 mM/600 mM) and passed a quality control (QC) analysis prior to the injection. A 243.2 ± 13.6‐mM HP pyruvate solution (pH 7.7 ± 0.4, 0.1 mmol/kg body weight) was injected at 5 mL/s, followed by a 25‐mL saline flush. Liquid‐state polarization was 34.3% ± 5.3% at the time of dissolution, with dissolution‐to‐injection time of 60.1 ± 12.4 s. Dissolution QC parameters are summarized in Table S1. Polarization levels were not available for participants #9–11 due to limitations of the updated QC system.

### 2.5 Image Reconstruction and Statistical Analysis

All ^13^C data were reconstructed and analyzed using MATLAB (MathWorks, Natick, MA). Reconstruction methods were based on previously described implementations for spCSI [9, 21], MISI [4], and MRS [3], with further details in the Supporting Information. Differences between injections were assessed by calculating the percent change in lactate‐to‐pyruvate ratio relative to the first injection.

Data are reported as mean ± standard deviation. Statistical analyses were performed using Prism 10 (GraphPad version 10.6.1). For blood, fractional ^13^C enrichments and total metabolite concentrations were compared to baseline (pre‐injection) using two‐tailed, paired *t*‐tests (*α* = 0.05). In addition, longitudinal blood measurements under fasted vs. post‐prandial states (i.e., nutritional conditions defined as overnight fast or after a meal) were compared using a two‐way repeated‐measures ANOVA with the Geisser–Greenhouse correction. Since participant #7 did not complete the fasting study, the participant's data were included only in the longitudinal fed group analysis and were excluded from the fasting group analysis and the fasted‐fed comparison.

For HP data, differences between consecutive injections were correlated with injection interval using Pearson's correlation analysis (*α* = 0.05). Measurement repeatability of time‐accumulated ^13^C‐lactate‐to‐pyruvate ratio in the brain was assessed using the intraclass correlation coefficient (ICC) based on a two‐way mixed‐effects model for absolute agreement of single measurements (ICC (3,1)). Bland–Altman analysis was also performed to evaluate agreement between consecutive measurements. For imaging data (spCSI and MISI), voxel‐wise Pearson's correlation and within‐subject coefficient of variation were also computed for each participant using lactate‐to‐pyruvate maps from the two injections. Brain masks were applied to exclude background. Pearson's correlation coefficient was calculated between corresponding voxel values to quantify spatial agreement between injections. The within‐subject coefficient of variation (wCV) was computed as follows:

$$\mathrm{wCV}=\frac{\left|X_{1}-X_{2}\right|}{\sqrt{2}\bar{\cdot X}}\times 100\;\left(\%\right)$$

where $X_{1}$ and $X_{2}$ denote voxel intensities from the two injections and $\bar{X}$ is their mean. The overall wCV for each participant was obtained by averaging voxel‐wise wCV values.

## 3 Results

**FIGURE 1:**
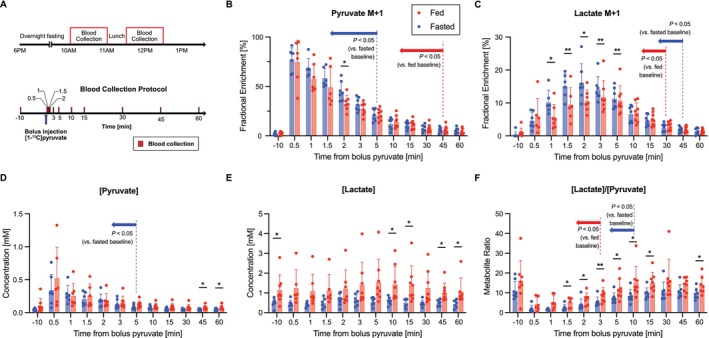
Changes of circulating pyruvate and lactate after bolus injection of [1‐^13^C]pyruvate under fasted and fed conditions. (A) Blood was collected from study participants (*n* = 8) immediately after bolus injection of non‐hyperpolarized [1‐^13^C]pyruvate under overnight fasted and postprandial conditions. (B) The fractional abundance of pyruvate M + 1 peaked at ∼30‐s post‐injection, and then gradually returned to the baseline. (C) The fractional abundance of labeled lactate rose more slowly, peaking at 2–3 min after injection, and then declined toward baseline. Although pyruvate enrichment showed no significant differences between conditions, lactate enrichment was higher in the fasted state compared with the fed state during the 1–5‐min post‐injection window. (D) Total plasma pyruvate concentration increased for the first 5 min in the fasted state. (E) Total lactate concentration was not altered by pyruvate bolus but remained higher in the fed state relative to the fasted state. (F) The lactate‐to‐pyruvate ratio decreased immediately following the pyruvate bolus and recovered within 5 min under fed conditions and 15 min under fasted conditions, with overall higher values observed in the fed state. *p < 0.05, **p < 0.01.

Study participants underwent bolus injections of thermal (non‐hyperpolarized) [1‐^13^C]pyruvate under both overnight fasted and postprandial conditions to assess how rapidly circulating pyruvate and lactate levels normalized (Figure 1A). Fractional enrichments of [1‐^13^C]pyruvate and [1‐^13^C]lactate (M + 1 isotopologues by mass spectrometry) changed significantly over time (*p* < 0.0001), with no significant effect of nutritional condition. However, there was a significant interaction between time and nutritional condition for lactate M + 1 enrichment (*p* = 0.047), whereas no interaction was observed for pyruvate M + 1. Pyruvate M + 1 enrichment peaked within 30 s of injection and decayed monotonically over the 60‐min observation window in both nutritional states (Figure 1B). Despite similar decay patterns between groups, pyruvate enrichment was no longer significantly different from baseline after 5 min in the fasted state but remained significantly elevated until 45 min in the fed state. Enrichment in lactate rose more gradually, reaching a maximum at 2 min (fasted) and 3 min (fed) post‐injection, then returned to baseline by 30 min in the fasted state and 45 min in the fed state (Figure 1C). While pyruvate enrichment dynamics were similar between conditions, lactate enrichment was significantly higher in the fasted state during the first 5 min after injection.

Total circulating pyruvate concentration increased transiently following the bolus and returned rapidly to baseline (Figure 1D; *p* = 0.0002), without nutritional effects. Circulating lactate levels did not change significantly following pyruvate injection, but overall concentrations were consistently higher in the fed state than in the fasted state (Figure 1E; *p* = 0.037). The lactate‐to‐pyruvate ratio decreased immediately after injection, reflecting the surge in pyruvate, and recovered within ∼10 min (Figure 1F). This ratio was overall higher in the fed condition than in the fasted condition. Blood glucose concentrations were 69.2 ± 26.2 mg/dL in the fasted state and 100.2 ± 55.5 mg/dL in the fed state (*p* = 0.045).

**FIGURE 2:**
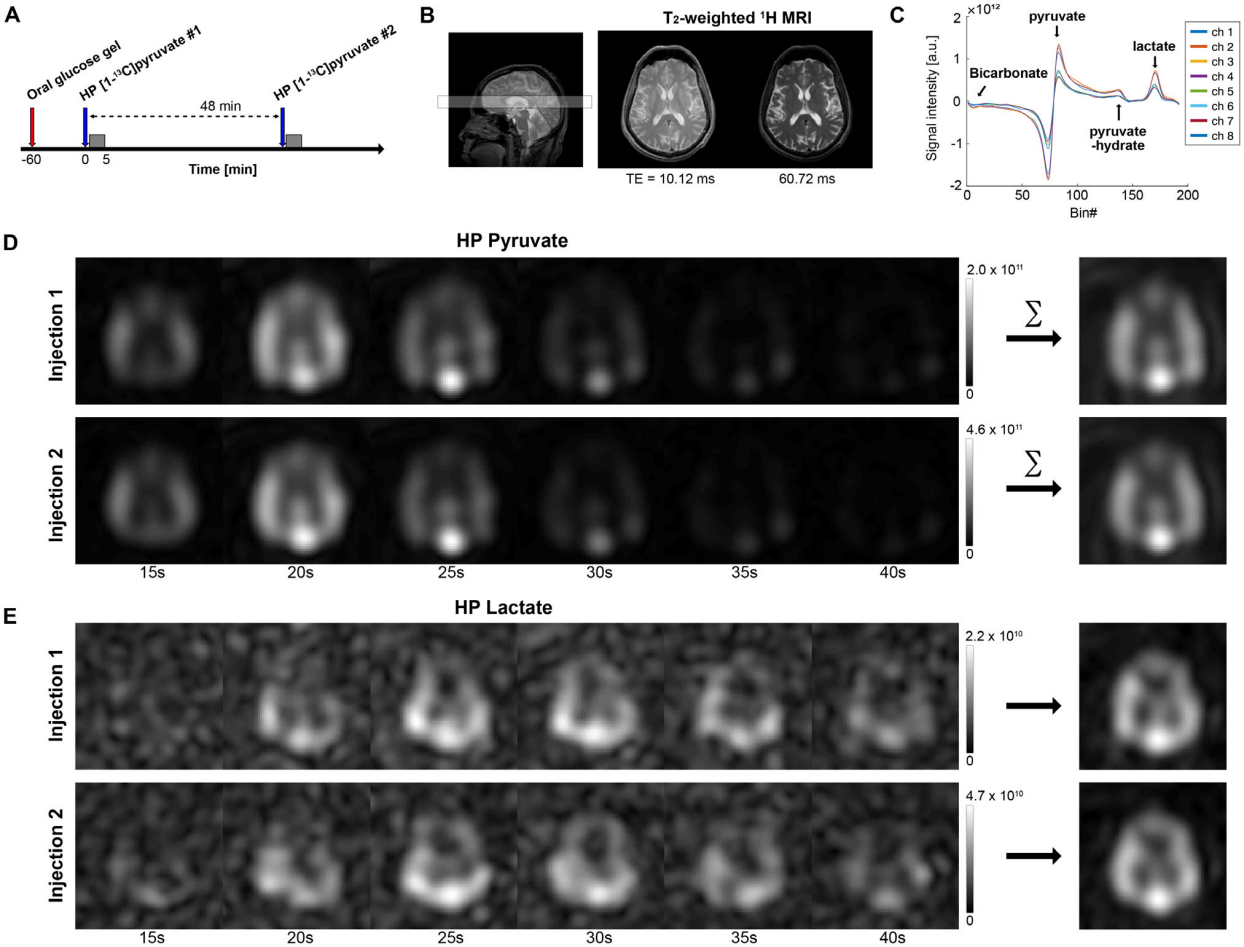
^13^C Spiral CSI of healthy volunteer with two consecutive HP pyruvate. (A) Two samples of HP [1‐^13^C]pyruvate were injected to a 63‐years‐old male (participant ID: #12) subjected for ^13^C spiral CSI with a 48‐min interval. (B) Prescribed axial slice for ^13^C imaging. (C) Averaged coil‐wise ^13^C spectra in pure absorption mode with 0th order phase correction for lactate peak. (D) Time‐resolved HP pyruvate and time‐accumulated pyruvate maps from each injection. (E) Time‐resolved HP lactate and time‐accumulated lactate maps.

**FIGURE 3:**
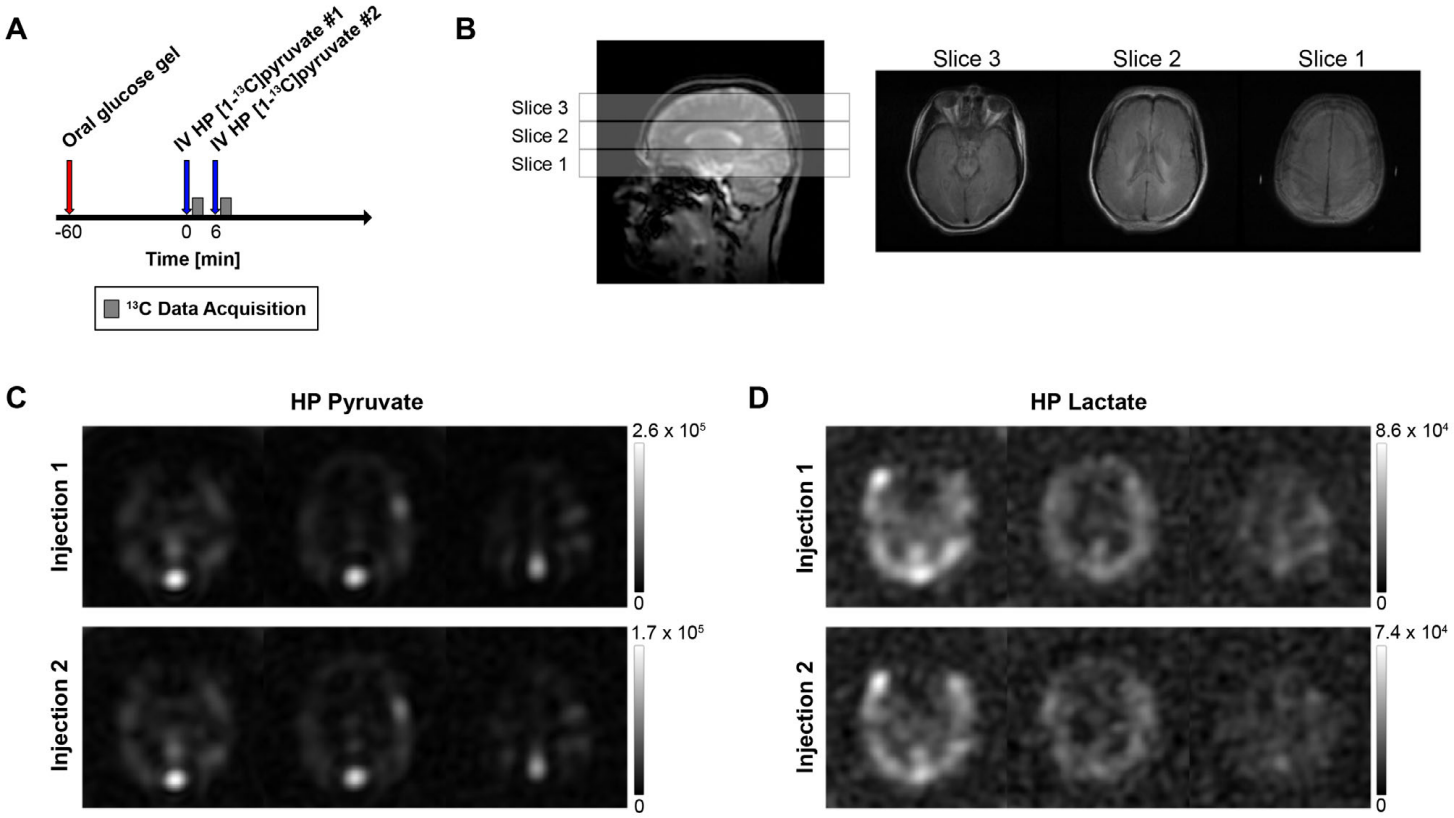
Metabolite‐interleaved spiral imaging of a healthy volunteer with two consecutive HP pyruvate. (A) Two samples of HP [1‐^13^C]pyruvate were injected to a 39‐years‐old male (participant ID: #9) subjected for ^13^C metabolite‐interleaved spiral imaging with a 6‐min interval. (B) ^1^H MRI of the prescribed axial slices for ^13^C imaging. Time‐accumulated ^13^C MRI of (C) HP pyruvate and (D) HP lactate maps from the corresponding slices.

Repeatability of HP [1‐^13^C]pyruvate MRI was evaluated using dynamic ^13^C spCSI and MISI. In a 63‐year‐old male (participant ID: #12), for example, dynamic profiles of HP pyruvate and HP lactate, as well as time‐averaged images acquired with spCSI, were comparable between two injections separated by 48 min, which is sufficient to allow circulating pyruvate and lactate to normalize (Figure 2). Although preliminary, results from a 39‐year‐old male (participant ID: #9) imaged with a shortened 6‐min reinjection interval using MISI also demonstrated comparable temporal dynamics across injections (Figure 3). The spatially distinct distributions of pyruvate and lactate in Figures 2 and 3 reflect their different compartmental contributions of pyruvate (vascular space) and lactate (brain tissue), consistent with previous brain studies using HP pyruvate [9, 22].

**FIGURE 4:**
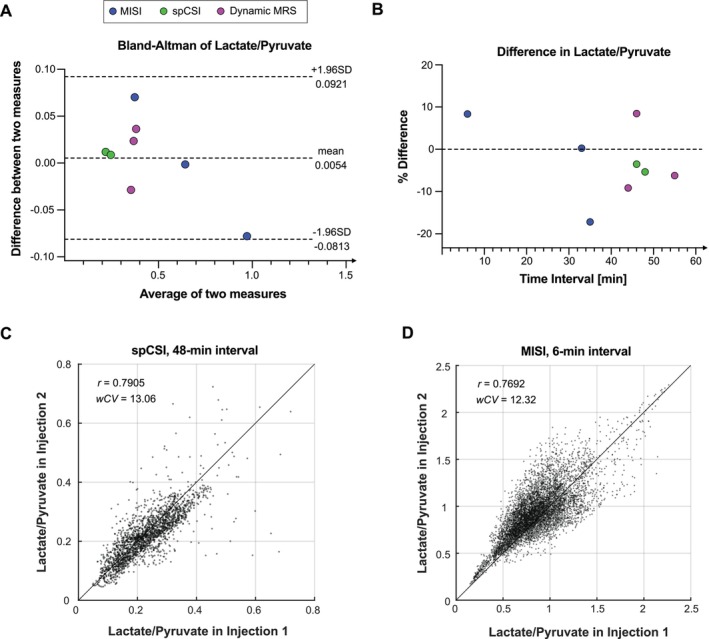
Repeatability and interval‐dependence of lactate‐to‐pyruvate ratios. (A) Difference versus average plot from the Bland–Altman analysis of two time‐accumulated HP lactate‐to‐pyruvate measurements in the brain supports repeatability of consecutive injections of HP [1‐^13^C]pyruvate. Horizontal dotted lines indicate upper (0.0921) and lower (−0.0813) limits of agreement. (B) Dependency of lactate‐to‐pyruvate ratio on the time interval between two pyruvate injections was not detected. Representative voxel‐wise scatter plots comparing lactate‐to‐pyruvate ratios from injection 1 and injection 2 for (C) spCSI with 48‐min interval (participant ID: #12) and (D) MISI with 6‐min interval (participant ID: #9) showed strong spatial agreement between injections. The solid line denotes the line of identity. MISI, metabolite‐interleaved spiral imaging; *r*, Pearson's correlation coefficient; spCSI, spiral CSI; wCV, within‐subject coefficients of variation.

Because absolute HP signals are influenced by polarization efficiency, in vivo T_1_ relaxation, and dissolution‐to‐injection time, direct quantification remains challenging. Lactate‐to‐pyruvate ratio maps provide a relative measure that mitigates these factors. Subtraction of lactate‐to‐pyruvate ratio maps from consecutive injections did not reveal noticeable differences with either spCSI or MISI (Figure S2). Time‐accumulated lactate‐to‐pyruvate measurements from all HP studies collectively demonstrated excellent repeatability (ICC (3,1) = 0.9842). Bland–Altman analysis showed a minimal mean bias of 0.0054 (standard deviation of bias = 0.0443), with 95% limits of agreement ranging from −0.0813 to 0.0921, confirming good agreement between repeated measurements (Figure 4A). Changes in the lactate‐to‐pyruvate ratio relative to the first injection showed no consistent trend with respect to injection interval (Figure 4B; *p* = 0.3301, *R* ^2^ = 0.1576).

For imaging data (ID: #9–13), voxel‐wise Pearson's correlation demonstrated moderate‐to‐high spatial agreement between injections (*r* = 0.72 ± 0.22). The wCV was 17.6% ± 4.6%, indicating overall good repeatability of metabolite measurements. Representative voxel‐wise scatter plots from the same participants from Figure 2 (ID: #12, spCSI, interval: 48 min) and Figure 3 (ID: #9, MISI, interval: 6 min) are shown in Figure 4C,D, respectively. Both examples demonstrate strong spatial agreement, with Pearson's correlation coefficients of *r* = 0.7905 and *r* = 0.7692. The corresponding wCV were 13.1% and 12.3%, respectively, indicating low relative variability between injections. The close alignment of data points along the identity line further supports the reproducibility of the measured metabolite signals across repeated acquisitions.

## 4 Discussion

In this study, we investigated the repeatability of HP [1‐^13^C]pyruvate in humans, with examples in neuroimaging. Longitudinal blood analysis showed that circulating pyruvate concentrations were elevated for ∼5 min following injection, whereas labeled lactate enrichment rose more gradually and remained significant for 30–45 min, despite total lactate concentration being unaffected. Importantly, the impact of a prior injection on subsequent imaging results was minimal, even with as short as a 6‐min interval between injections, suggesting that residual labeled metabolites in blood do not impose a major physiological limitation within this time frame. The 6‐min interval represents a technical lower bound in the current implementation and is dictated by the SPINlab system, which requires 5 min between dissolutions and an additional 1 min to accommodate QC procedures and dissolution‐to‐injection timing. Shorter inter‐scan intervals may be feasible in future systems with optimized polarization and delivery pipelines.

The rationale for analyzing circulating pyruvate and lactate is that they influence the cerebral metabolism observed with HP ^13^C imaging. In addition to label exchange rates between HP ^13^C‐pyruvate/^13^C‐lactate and endogenous metabolite pools [14], variations in their pool sizes can affect transport across the blood–brain barrier (BBB) [23]. The BBB regulates the exchange of both pyruvate and lactate between blood and brain tissue via monocarboxylate transporters, with substrate affinities that differ among brain cell types [24, 25, 26]. Indeed, multiple brain studies have shown that apparent HP pyruvate‐to‐lactate conversion rates are affected by BBB transport and delivery [27, 28, 29, 30]. Consequently, variations in circulating metabolite levels may alter both the pool sizes and substrate delivery across the BBB, thereby influencing the metabolic signals detected with HP ^13^C imaging.

The rapid stabilization of total lactate concentration in the current study, despite the sustained [^13^C]lactate enrichment, is consistent with robust systemic regulation of lactate homeostasis [31], whereby infusion of labeled substrate leads to durable isotopic enrichment without measurably perturbing the endogenous lactate pool. Although small changes in total lactate concentration cannot be entirely excluded due to inter‐individual variability, the persistent ^13^C enrichment likely reflects ongoing isotopic exchange rather than a sustained increase in total lactate.

Comparable findings have been reported with two consecutive injections of HP pyruvate in cardiac and cancer studies. Repeated HP pyruvate injections yielded consistent whole‐heart MRS measurements of cardiac metabolism [10] despite numerous physiological factors such as heart rate, coronary blood flow, cardiac phase, nutritional state, and motion artifacts [32]. Repeatability of HP pyruvate was also reported in pilot studies with patients with prostate cancer [6], renal tumors [7], and kidney transplant [5]. The current study focused on the brain because of its stability with respect to motion, positioning, and carbohydrate metabolism, making it a suitable organ for assessing repeatability based on lactate clearance.

The presence of residual ^13^C‐labeled metabolites beyond lactate and pyruvate was not systematically evaluated in this study, primarily due to their limited detection sensitivity in the brain. For instance, [1‐^13^C]alanine is produced predominantly in peripheral tissues, and cerebral alanine signals remain below the detection threshold within the HP acquisition window [26, 33]. [^13^C]Bicarbonate, the primary mitochondrial product via neuronal pyruvate dehydrogenase in the brain [26], has been reported in several human studies [3, 11, 22, 34, 35], but showed inconsistent SNRs across acquisition methods in the current work. However, the repeatability of imaging mitochondrial products has been demonstrated in preclinical neuroimaging studies, in which HP pyruvate doses (0.5–1.5 mmol/kg) exceeded typical human doses [36, 37]. In these studies, consecutive injections separated by 1–1.5 h, permitting clearance of circulating ^13^C‐pyruvate and ^13^C‐lactate, showed minimal differences in [^13^C]bicarbonate and [5‐^13^C]glutamate derived from HP [1‐^13^C]pyruvate and HP [2‐^13^C]pyruvate, respectively.

The current study has several limitations that suggest directions for future work. First, physiological factors beyond blood chemistry were not systematically monitored. For instance, changes in brain orientation and position between injections were not verified with additional ^1^H imaging. Second, diverse acquisition schemes were employed in HP studies. Differences in acquisition strategies, including RF excitation schemes, temporal sampling patterns, and spatial coverage, may influence the variability of the measured metabolite ratios. For instance, variations in acquisition delay and flip angle scheme can introduce metabolism‐weighted [3] and saturation‐related biases [38], potentially contributing to variability in lactate‐to‐pyruvate ratios. A systematic evaluation of these methodological factors would require dedicated simulations and studies under strictly controlled experimental settings. Third, HP repeatability was assessed in a small sample, and therefore, larger studies are needed to confirm these findings, particularly for short‐interval imaging protocols. Consistent with these considerations, the relatively large variation in the lactate‐to‐pyruvate ratio observed with R may reflect method‐specific susceptibility to physiological and experimental factors.

## 5 Conclusion

We demonstrate that intravenously injected ^13^C‐pyruvate clears from the circulation within 5 min. Although elevated ^13^C‐lactate enrichment persists longer, total lactate concentration in blood was unchanged by pyruvate injection. Consecutive hyperpolarized pyruvate injections produced highly comparable pyruvate, lactate, and lactate‐to‐pyruvate maps, with excellent repeatability within a single MR session. Collectively, our results demonstrate that hyperpolarized [1‐^13^C]pyruvate metabolic imaging is repeatable within a single session and that residual circulating ^13^C enrichment does not compromise quantitative metabolic readouts across consecutive injections.

## Funding

This work was supported by US Army Medical Research Acquisition Activity, HT94252510616, W81XWH2210485; National Center for Research Resources, S10RR029119; National Institute of Biomedical Imaging and Bioengineering, P41EB015908; National Institute of Diabetes and Digestive and Kidney Diseases, P30DK127984; NIH Office of the Director, S10OD028490; National Institute of Neurological Disorders and Stroke, R01NS107409; National Heart, Lung, and Blood Institute, R01HL170039.

## Supporting information


**Figure S1:** ^13^C Acquisition methods. (A) ^13^C/^1^H dual‐frequency RF head coil. (B) Overall schemes of dynamic acquisition methods. MR pulse sequence diagrams for (C) spiral CSI, (D) metabolite‐interleaved spiral imaging, and (E) MRS.
**Figure S2:** Changes in lactate‐to‐pyruvate ratios. Time‐accumulated lactate‐to‐pyruvate ratio maps from the study participants, shown in Figure 3 and Figure 4, using (A) spiral CSI and (B) metabolite‐interleaved spiral imaging, respectively.
**Table S1:** HP Parameters.

## Data Availability

The data that support the findings of this study are available on request from the corresponding author. The data are not publicly available due to privacy or ethical restrictions.
