# Supplementary material for: Repeatability of Metabolic Imaging With Hyperpolarized Pyruvate: Back‐to‐Back Neuroimaging and Blood Analysis
Source: Magn Reson Med. 2026 Apr 23;96(2):548–56. doi: 10.1002/mrm.70402 (PMC13269210; doi:10.1002/mrm.70402)
Supplement: Supplementary file 1 — Figure S1: 13C Acquisition methods. (A) 13C/1H dual‐frequency RF head coil. (B) Overall schemes of dynamic acquisition methods. MR pulse sequence diagrams for (C) spiral CSI, (D) metabolite‐interleaved spiral imaging, and (E) MRS. Figure S2: Changes in lactate‐to‐pyruvate ratios. Time‐accumulated lactate‐to‐pyruvate ratio maps from the study participants, shown in Figure 3 and Figure 4, using (A) spiral CSI and (B) metabolite‐interleaved spiral imaging, respectively. Table S1: HP Parameters. [file MRM-96-548-s001.docx]

**SUPPORTING INFORMATION**

**Title**: Repeatability of metabolic imaging with hyperpolarized pyruvate: back-to-back neuroimaging and blood analysis

**Authors**: Jun Chen, Ph.D.^a,†^, Sung-Han Lin, Ph.D.^a,†^, Jessica Sudderth, Ph.D.^b^, Kelley A. Derner, R.N.^a^, Crystal E. Harrison, Ph.D.^a^, Maheen Zaidi, B.S.^a^, Jeannie D. Baxter, R.N.^a^, Jeff Liticker, Pharm.D.^a^, Zohreh Erfani, M.D.^a^, Craig R. Malloy, M.D.^a,c^, Marco C. Pinho, M.D.^c^, Ralph J. DeBerardinis, M.D., Ph.D.^b,d,e^, Jae Mo Park, Ph.D.^a,c,f,g^*

**Affiliations**:

1. Advanced Imaging Research Center, The University of Texas Southwestern Medical Center, Dallas, TX, USA
2. Children’s Research Institute, The University of Texas Southwestern Medical Center, Dallas, TX, USA
3. Department of Radiology, The University of Texas Southwestern Medical Center, Dallas, TX, USA
4. Howard Hughes Medical Institute, Dallas, TX, USA
5. McDermott Center for Human Growth and Development, The University of Texas Southwestern Medical Center, Dallas, TX, USA
6. Department of Biomedical Engineering, The University of Texas Southwestern Medical Center, Dallas, TX, USA
7. Charles and Jane Pak Center for Mineral Metabolism and Clinical Research, The University of Texas Southwestern Medical Center, Dallas, TX, USA

† indicates equal contribution.

***Correspondence**: Jae Mo Park, Ph.D.

5323 Harry Hines Boulevard, Dallas, Texas, United States, 75390-8568

Email: [jaemo.park@utsouthwestern.edu](mailto:jaemo.park@utsouthwestern.edu), Tel: +1-214-645-7206, Fax: +1-214-645-2744

**Supplemental Methods**

MR Protocols

A rigid nested design was applied to the ^1^H coil (∅ [diameter] = 30.4 cm, length = 30.5 cm) and the ^13^C transmit coil (∅ = 26.8 cm, length = 25.4 cm). The anterior ^13^C receive array (5-8 channels) are attached to the inner surface of the ^13^C transmit coil, and the posterior array (1-4 channels) are flexible to maximize the receive sensitivity. Each coil element of the ^13^C Rx arrays was 12.7 cm × 16.5 cm. The entire anterior portion of the head coil is detachable for head positioning. The coil performance was reported previously (1).

^1^H MRI was performed first with a fast GRE localizer and a 2D T_2_-weighted fluid-attenuated inversion recovery (FLAIR) to localize the brain and prescribe imaging slices. Three different time-resolved acquisition schemes were exploited for ^13^C acquisition. Dynamic spiral chemical shift imaging (CSI) was applied with field-of-view (FOV) = 24 cm × 24 cm, matrix size = 16 × 16, #slice = 1, slice thickness = 3 cm, variable flip angle up to 30^o^ per timepoint, temporal resolution = 5 sec, seven spatial interleaves of spiral readout, 48 echoes, and spectral width = 814.3 Hz. Dynamic, metabolite-interleaved spiral imaging was applied with FOV = 24 cm × 24 cm, matrix size = 16 × 16, #slice = 3, slice thickness = 3 cm, single shot, flip angle = 90^o^ for bicarbonate, 90^o^ for lactate, 10^o^ for pyruvate, #echoe = 5/metabolite, and temporal resolution = 5 sec using previously designed spectral-spatial RF pulse (2). Dynamic MRS was used with flip angle = 6.5^o^, temporal resolution = 3 sec, spectral width = 10000 Hz, spectral points = 4096, #slice = 4, and slice thickness = 1.5 cm. The transmit and receive ^13^C frequencies were calculated from the ^1^H water frequency of the PRESS sequence and set on [1-^13^C]pyruvate resonance (3). Additional ^1^H images such as dual echo T_2_-weighted fast spin echo (FSE; TE = 10.12 ms/60.72 ms) were acquired during the time interval.

Preparation, Polarization, and Polarization of Pyruvic Acid

Two pyruvate samples were simultaneously polarized for each subject using a SPINlab DNP polarizer. Each pyruvate sample was prepared by mixing 1.47 g of GMP-grade 14-M [1-^13^C]pyruvic acid (Sigma Aldrich, St Louis, Missouri, USA) with 27.7 mg of AH111501 electron paramagnetic agent (EPA) radical (Syncom, Groningen, Netherlands). After assembled in a sterile environment, clinical fluid paths were equipped to the SPINlab for polarization. The pyruvate samples were polarized for 3-4.5 hours, then dissolved by 38 mL of sterile water at 130 ^o^C. The HP pyruvate solution was mixed with 36.5 mL of room temperature TRIS/NaOH media (333 mM/600 mM) and passed a QC analysis prior to the injection. Terminal filtering, bubble point test, pH strip confirmation and volume check were performed after the automated QC.

Reconstruction and Data Processing

Data acquired with spiral CSI was apodized using a 10-Hz Gaussian filter, zero-filled by a factor of four, and fast Fourier transformed along the echo domain. After gridding the data onto k-space, the data were spatially apodized using a Hanning filter (α = 0.66), zero-filled by a factor of four, and 2D inverse Fourier transformed along *k_x_* and *k_y_*. Each coil data was combined with phase-office correction, then each metabolite peak was measured in pure absorption mode with 0^th^ order phase correction for the corresponding resonance. The resulting dynamic metabolite maps were added to create time-accumulated maps. Data acquired with metabolite interleaved spiral imaging was processed similarly, excluding the initial steps along the echo domain. MRS was reconstructed by performing apodization using a 5-Hz Gaussian filter, four-fold zero-filling, and fast Fourier transform along the FID. For combining two data sets, the raw data were added before the reconstruction procedure. Lactate-to-pyruvate ratio maps were created by pixelwise division. For display purpose, a threshold was applied to avoid large background noise in the ratio maps.

**REFERENCES**

1. Ma J, Pinho MC, Harrison CE, et al. Dynamic (13) C MR spectroscopy as an alternative to imaging for assessing cerebral metabolism using hyperpolarized pyruvate in humans. Magn Reson Med 2022;87(3):1136-1149.

2. Ma J, Chen J, Reed GD, et al. Cardiac T2 * measurement of hyperpolarized (13) C metabolites using metabolite-selective multi-echo spiral imaging. Magn Reson Med 2021;86(3):1494-1504.

3. Park JM, Harrison CE, Ma J, et al. Hyperpolarized (13)C MR Spectroscopy Depicts in Vivo Effect of Exercise on Pyruvate Metabolism in Human Skeletal Muscle. Radiology 2021;300(3):626-632.

**Supplemental Figures**

**Figure S1. ^13^C Acquisition methods.** (A) ^13^C/^1^H dual-frequency RF head coil. (B) Overall schemes of dynamic acquisition methods. MR pulse sequence diagrams for (C) spiral CSI, (D) metabolite-interleaved spiral imaging, and (E) MRS.

**Figure S2. Changes in lactate-to-pyruvate ratios.** Time-accumulated lactate-to-pyruvate ratio maps from the study participants, shown in **Figure 3** and **Figure 4**, using (A) spiral CSI and (B) metabolite-interleaved spiral imaging, respectively.

**Supplemental Tables**

**Table S1. HP Parameters**

| Participant ID | Dissolution order | Polarization time [min] | Dissolution-to-injection time [sec] | Liquid-state polarization level [%] | Pyruvate concentration [mM] | pH | EPA concentration [μM] | Temperature [^o^C] |
| --- | --- | --- | --- | --- | --- | --- | --- | --- |
| 9 | 1 | 240 | 47 | - | 241 | 8.0 | 0.9 | 26 |
|  | 2 | 229 | 45 | - | 249 | 8.0 | 0.4 | 23 |
| 10 | 1 | 198 | 54 | - | 254 | 6.6 | 3.7 | 30 |
|  | 2 | 182 | 49 | - | 242 | 7.0 | 4.0 | 29 |
| 11 | 1 | 207 | 48 | - | 260 | 7.5 | 0.8 | 29 |
|  | 2 | 192 | 44 | - | 247 | 7.6 | 1.1 | 29 |
| 12 | 1 | 245 | 75 | 39 | 221 | 8.2 | 0.7 | 29.5 |
|  | 2 | 224 | 80 | 37 | 234 | 7.9 | 1.4 | 35.5 |
| 13 | 1 | 171 | 78 | 38 | 242 | 8.0 | 1.7 | 32.9 |
|  | 2 | 181 | 80 | 41 | 234 | 7.9 | 1.6 | 33.0 |
| 14 | 1 | 214 | 62 | 41 | 266 | 7.5 | 0 | 34.2 |
|  | 2 | 172 | 61 | 39 | 255 | 7.9 | 0.3 | 35.8 |
| 15 | 1 | 228 | 61 | 28 | 240 | 7.5 | 0.8 | 36.4 |
|  | 2 | 211 | 59 | 27 | 217 | 8.1 | 0.5 | 33.2 |
| 16 | 1 | 263 | 62 | 33 | 233 | 7.8 | 2.5 | 33.8 |
|  | 2 | 202 | 59 | 29 | 236 | 7.9 | 0.5 | 34.2 |
